# Bio-Inspired Stretchable Absolute Pressure Sensor Network

**DOI:** 10.3390/s16010055

**Published:** 2016-01-02

**Authors:** Yue Guo, Yu-Hung Li, Zhiqiang Guo, Kyunglok Kim, Fu-Kuo Chang, Shan X. Wang

**Affiliations:** 1 Department of Electrical Engineering, Stanford University, 350 Serra Mall, Stanford, CA 94305, USA; kyunglok.kim@gmail.com (K.K.); sxwang@stanford.edu (S.X.W.); 2 Department of Materials Science and Engineering, Stanford University, 476 Lomita Mall, Stanford, CA 94305, USA; liyuhung@stanford.edu; 3 Department of Mechanical Engineering, Stanford University, 440 Escondido Mall, Stanford, CA 94305, USA; zguo@stanford.edu; 4 Department of Aeronautics and Astronautics, Stanford University, 496 Lomita Mall, Stanford, CA 94305, USA; fkchang@stanford.edu

**Keywords:** stretchable network, absolute pressure sensor, MEMS, smart skin

## Abstract

A bio-inspired absolute pressure sensor network has been developed. Absolute pressure sensors, distributed on multiple silicon islands, are connected as a network by stretchable polyimide wires. This sensor network, made on a 4’’ wafer, has 77 nodes and can be mounted on various curved surfaces to cover an area up to 0.64 m × 0.64 m, which is 100 times larger than its original size. Due to Micro Electro-Mechanical system (MEMS) surface micromachining technology, ultrathin sensing nodes can be realized with thicknesses of less than 100 µm. Additionally, good linearity and high sensitivity (~14 mV/V/bar) have been achieved. Since the MEMS sensor process has also been well integrated with a flexible polymer substrate process, the entire sensor network can be fabricated in a time-efficient and cost-effective manner. Moreover, an accurate pressure contour can be obtained from the sensor network. Therefore, this absolute pressure sensor network holds significant promise for smart vehicle applications, especially for unmanned aerial vehicles.

## 1. Introduction

Recently, bio-inspired multifunctional sensor networks, the fundamental component of smart artificial skin, have attracted increasing attention due to their emerging broad application perspectives [1,2]. A variety of sensor networks with different sensors to detect physical parameters, such as temperature, strain, and vibration, have been studied [3,4,5]. Absolute pressure is also one useful parameter to be tracked, especially in the fields of automotive, nautical, and aerospace applications, where next-generation self-aware smart vehicles rely heavily on accurate absolute pressure sensing [6,7,8,9]. In the case of Unmanned Aerial Vehicles (UAVs), monitoring the absolute pressure distribution surrounding the entire aircraft in real-time is highly desirable for assessing its health condition and adjusting itself to avoid hazardous stalling [10]. Thus, an absolute pressure sensor network consisting of a large amount of sensing elements with high sensitivity is required for the purpose of achieving adequate sensing resolution. The sensor network also needs flexibility to be mounted on curvature surfaces, such as an airfoil in smart autonomous vehicle applications. Additionally, small size, light weight, and low cost are highly preferred due to practical application needs.

Micro Electro-Mechanical System (MEMS) sensors with piezoresistive and capacitive pressure sensing mechanisms have been widely explored in previous studies, not only on rigid silicon wafers but different flexible substrates as well [11,12,13,14,15,16,17]. Although high sensitivity can be achieved in traditional MEMS pressure sensors, those sensors, generally built on rigid silicon substrates, do not feature useful stretchability and flexibility. In this work, a stretchable absolute pressure sensor network has been developed, aimed at application in smart autonomous vehicles, with decision-making capability and self-adaptive controllability. Absolute pressure sensors are located on multiple silicon islands and connected as a network by routing aluminum wires supported by flexible stretchable straps made of polyimide. This sensor network, originally fabricated on a 4’’ silicon wafer, can be expanded up to 0.64 m × 0.64 m, which is 100 times larger than its original size. In terms of transducer mechanisms, piezoresistance is used to guarantee performance of sensitivity and linearity, as well as low complexity and cost. Thanks to MEMS surface micromachining technology, very thin pressure sensors can be fabricated on Silicon-On-Insulator (SOI) wafers. Since the MEMS sensor fabrication process has been well integrated with a flexible polymer substrate process, the entire sensor network can be made efficiently in one process on standard silicon wafers, then subsequently stretched, and finally mounted on various surfaces, where no further sensor alignment and interconnection are needed. Furthermore, an accurate pressure contour can be achieved from a sufficient number of sensing nodes, and a self-awareness intellectual system can be improved by taking advantages of absolute pressure distribution data captured from entire vehicle surfaces. Therefore, this new pressure sensor network holds significant promise for smart vehicle applications.

**Figure 1:**
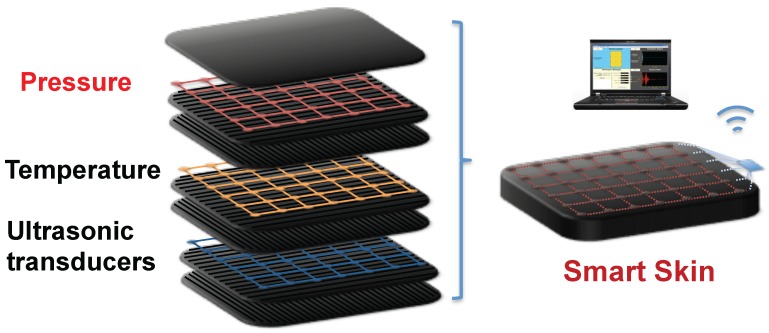
Schematic overview of stretchable pressure sensor network integrated into smart skin materials.

Figure 1 illustrates a schematic overview of smart skin materials in smart vehicle applications. The stretchable pressure sensor network, along with other types of networks such as temperature and vibration, can be integrated into a smart skin. In order to sense the absolute pressure, this pressure sensor network needs to be placed near the top surface of the smart skin structure. Voltage signals measured from the pressure network can be collected locally and sent to a central controller by a wireless transmitter.

## 2. Experimental Section

**Figure 2:**
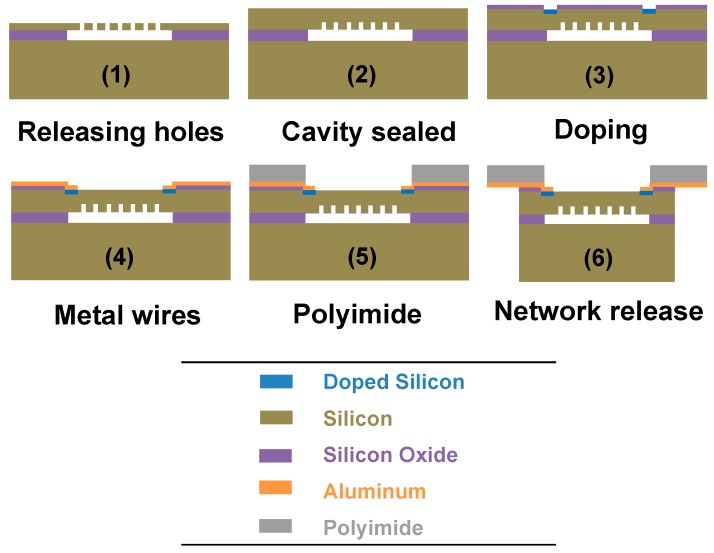
Main fabrication processes of the stretchable pressure sensor network.

A fabrication process, combining surface micromachining and a flexible polymer substrate process together, has been developed to realize the stretchable pressure sensor network. Figure 2 illustrates the main fabrication steps. The piezoresistive absolute pressure sensing elements include a sealed vacuum cavity, a single-crystal silicon diaphragm in a square shape, and top electrodes. An SOI wafer is used as a starting substrate with a 2 μm-thick buried oxide layer. Multiple tiny holes with a diameter of 0.6 μm, which serve as venting holes, are first etched through the top silicon device layer using deep reactive-ion etching (DRIE). The buried oxide layer is then etched by a subsequent vapor-phase hydrofluoric acid (HF) process for 5 h, to release the top active layer. Next, these venting holes are sealed by the growth of single-crystal silicon at 1150 °C in an Applied Materials Centura epitaxial system. A high-quality single-crystal silicon membrane is preferred for obtaining large piezoresistivity, in order to achieve a sensitive and linear response to pressure variations. The silicon diaphragm has a square shape with a dimension of 550 μm by 550 μm. After desired mask patterns are defined lithographically, ion implantation of boron and drive-in annealing are performed to change active silicon areas to *p*-type silicon with a boron doping concentration of ~5 × 10^18^ cm^−3^. By optimizing process parameters, a good electrical insulation is achieved between the active piezoresistors and the *n*-type substrate.

The steps discussed above are suitable for building high-performance sensing elements, whereas the following steps serve to connect elements as a stretchable sensor network. An aluminum layer, which serves as electrodes and wires, is first sputtered on top of the diaphragm. Next, a polyimide layer of 15 μm thickness is spin coated onto the aluminum layer, followed by another aluminum deposition as a mask layer. The stretchable wires are then lithographically patterned and dry etched by oxygen plasma. Finally, the device wafer is mounted upside down onto another carrier wafer, and the process concludes with a through silicon etch by DRIE to release the sensor network. Therefore, the MEMS absolute pressure sensors can be fully integrated with the stretchable polyimide substrate.

## 3. Results and Discussion

**Figure 3:**
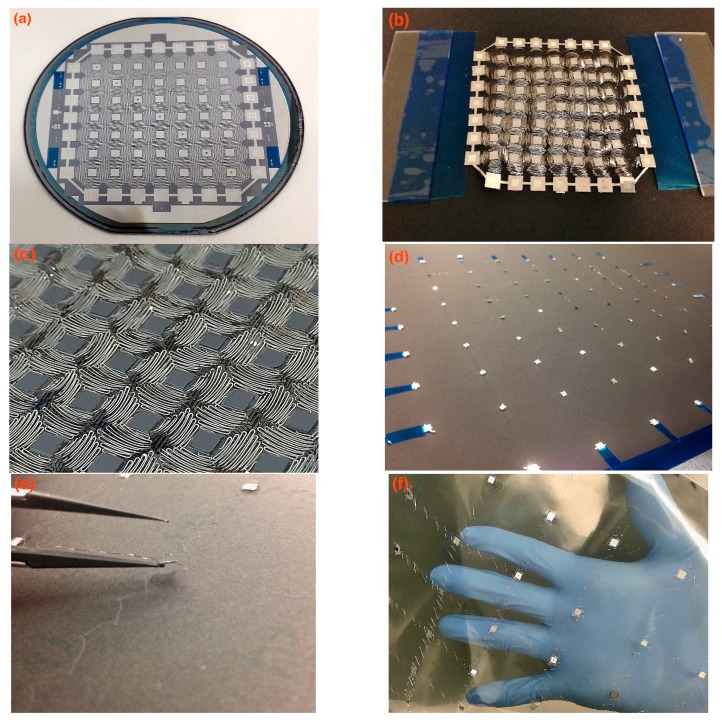
(**a**) Patterned pressure sensor network before releasing; (**b**) Released network before stretching; (**c**) Zoomed-in view of stretchable wires; (**d**) Stretched pressure sensor network; (**e**) Zoomed-in view of a silicon island and stretchable wires; (**f**) Stretched pressure network mounted onto a PVC soft film.

In Figure 3, the stretchability and flexibility of the absolute pressure sensor network is demonstrated. Figure 3a shows a patterned pressure sensor network on a 4’’ silicon wafer before release. There are seven pressure-sensing elements on this network, located on silicon islands aligned in the diagonal direction, whereas the remaining islands are used only for routing signals out in this demonstration with a single layer of aluminum wire. Further, more pressure sensing nodes can be available on a network with multilayer aluminum wires. Figure 3b depicts a released network. Small anchors are adopted to hold together the silicon islands at the edges. Figure 3c is a zoomed-in view of stretchable wires and silicon islands from the backside, which illustrates the good condition of wires after releasing. The polyimide wires are robust enough to hold the silicon islands that have a dimension of 4 mm by 4 mm, and each individual wire connects two islands. An expanded pressure sensor network is shown in Figure 3d. It has 77 nodes and covers an area up to about 0.64 m × 0.64 m, which is 100 times larger than its original size. Sensing elements are located at the center of rigid silicon islands, with a sealed vacuum cavity for the purpose of absolute pressure sensing. Due to the rigidity of individual silicon nodes, performances of individual absolute pressure sensing elements on the stretchable network are similar to separate micromachined transducers. A closer view of the island is given in Figure 3e. By leveraging the benefits from MEMS surface micromachining process, 4 mm × 4 mm silicon islands can be thinned down to less than 100 µm. Compared with typical commercial MEMS pressure sensors that are usually made by bulk micromachining technology, the ultrathin sensing elements make them well suited for installation on vehicle surfaces without affecting the airflow. In Figure 3f, a stretched pressure network is mounted onto a PVC soft film surface, and is easily held by hand. As shown, the proposed stretchable absolute sensor network holds great potential to be mounted onto various surfaces in integration.

In terms of transducer mechanisms, both piezoresistive and capacitive sensing approaches can be used on the stretchable absolute pressure sensor network. Table 1 lists characteristic performance metrics for comparison between piezoresistive and capacitive sensing mechanisms [13,18,19,20]. Compared with capacitive sensing, piezoresistive mechanism has three advantages when applied to stretchable absolute pressure sensor networks: excellent linearity, simple interface electronics, and low cost. Firstly, an excellent linearity can typically be achieved on the network sensing nodes with small diaphragms. By contrast, capacitance changes nonlinearly with diaphragm displacement and its corresponding applied pressure in a capacitive absolute pressure sensor with parallel plates. Secondly, a piezoresistive absolute pressure sensor has relatively simple interface electronics, whereas a capacitive pressure sensor typically requires additional interface electronics to convert the sensor capacitance value to a voltage output. Thirdly, piezoresistive pressure sensors have fewer lithography steps in their fabrication process, thereby greatly reducing their complexity and cost. In the case of capacitive sensors, both top and bottom electrodes are needed, complicating the micromachining process. Because of these benefits discussed above, the piezoresistive transducer mechanism is employed in this work to construct the stretchable absolute pressure sensor network.

**Table 1:** Comparison between piezoresistive and capacitive sensing mechanisms.

Characteristic	Piezoresistive Sensing	Capacitive Sensing
Linearity	Good	Fair
Accuracy	±1%	±0.2%
Resolution	1 part in 10^5^	1 part in 10^4^ to 10^5^
Temperature error	~1600 × 10^−6^/°C	~4 × 10^−6^/°C
Cost	Low	Medium
Electronics	Simple	Complex

**Figure 4:**
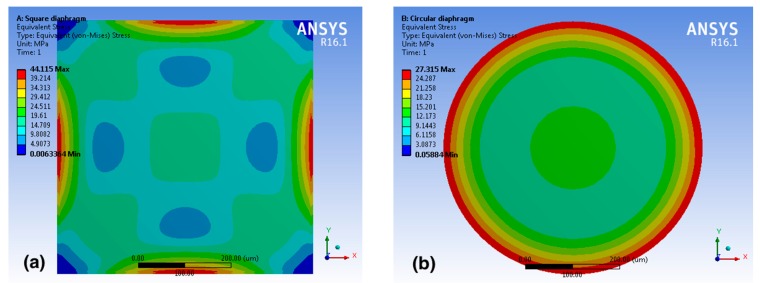
Contour plot of the Von Mises stress distribution on (**a**) a square and (**b**) a circular diaphragm.

In order to understand the mechanical characteristics of the sensing elements, both analytical calculations and multi-physics finite element analysis were conducted [21,22,23]. When a pressure difference exists between the inside and outside of the cavity, the top silicon diaphragm deforms correspondingly. Figure 4 plots finite element simulation results from the Ansys software.

**Figure 5:**
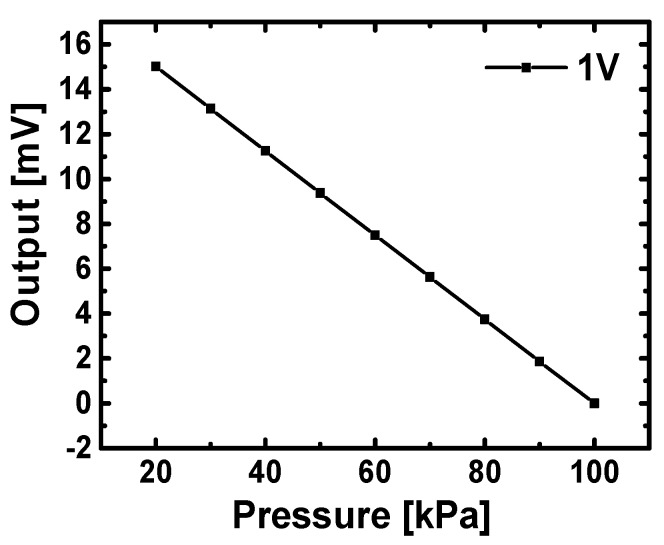
Simulated results of a pressure sensing element at 1 V input supply.

The color contours illustrate Von Mises stress distribution in MPa on a square and a circular diaphragm, respectively. Both square and circular diaphragms have the same thickness and edge/diameter length. In comparison with circular diaphgrams, square diaphragms show better performance. When a uniform pressure of 100 kPa is applied, the maximum stress of the square diaphragm is about 1.62 times as large as that of the circular diaphragm, which is also consistent with prior results in literature [24]. Therefore, square diaphragms are employed in sensing elements of the stretchable sensor network. Based on our simulation results, maximum stresses occur near the edges of the square diaphragm. Hence, four piezoresistors are patterned at the most sensitive regions, and two different pairs of piezoresistor geometries are adopted at the opposite edges to increase the sensitivity. Also, sensors are connected in a full Wheatstone bridge configuration, which enables differential sensing and helps reduce errors from temperature changes. Assuming the full Wheatstone bridge is well balanced, and all four piezoresistors have the same resistance value, the relative output voltage change of the sensing elements can be expressed as follows [25]:

(1) $$\frac{∆V}{V}=\frac{∆R}{R}=\pi_{l}\sigma_{l}+\pi_{t}\sigma_{t}$$

where π_l_ and σ_l_ are the piezoresistive coefficient and stress in the longitudinal direction of piezoresistors, and π_t_ and σ_t_ are the values in the transverse direction. In order to achieve optimum sensitivity, the piezoresistors are aligned along the <110> direction on a p type (100) silicon wafer. Since piezoresistive coefficient is highly dependent on doping concentration [12,19], it drops to about 70% of its maximum value at a doping concentration of ~5 × 10^18^ cm^−3^. Figure 5 shows simulated results of a pressure sensing element at 1V input supply, and illustrates that a linear voltage output with a sensitivity of 18.7 mV/V/bar can be expected from sensing elements on the stretchable pressure sensor network.

**Figure 6:**
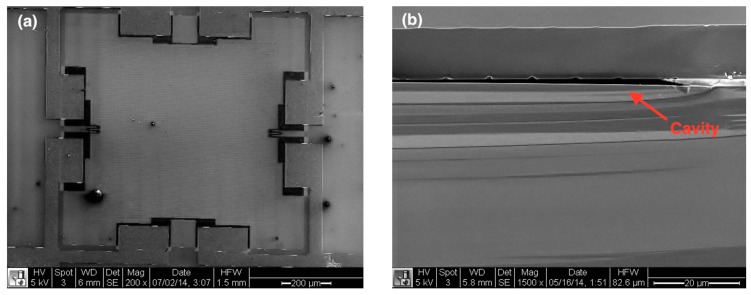
(**a**) Top view of a piezoresistive pressure sensing element by SEM; (**b**) SEM cross-section view of a sensing element.

Figure 6a is a scanning electron microscopy (SEM) top-view of the MEMS absolute pressure transducer for the stretchable sensor network. The dark black regions near the edges of the top silicon square diaphragm in the plot are four piezoresistors with a concentration of boron doping (~5 × 10^18^ cm^−3^), which gives a resistance value about 2 kΩ. The doping concentration was optimized not only to achieve high piezoresistivity but also for good isolation. Furthermore, an aluminum layer, which connects those piezoresistors to each other via ohmic contact, is deposited at the same time as routing wires for the sensor network. Figure 6b provides an SEM cross-section view showing a cavity sealed by a single-crystal silicon membrane. This high-quality top membrane, epitaxially grown at a high temperature of 1150 °C, offers high piezoresistivity, which then confers high sensitivity.

**Figure 7:**
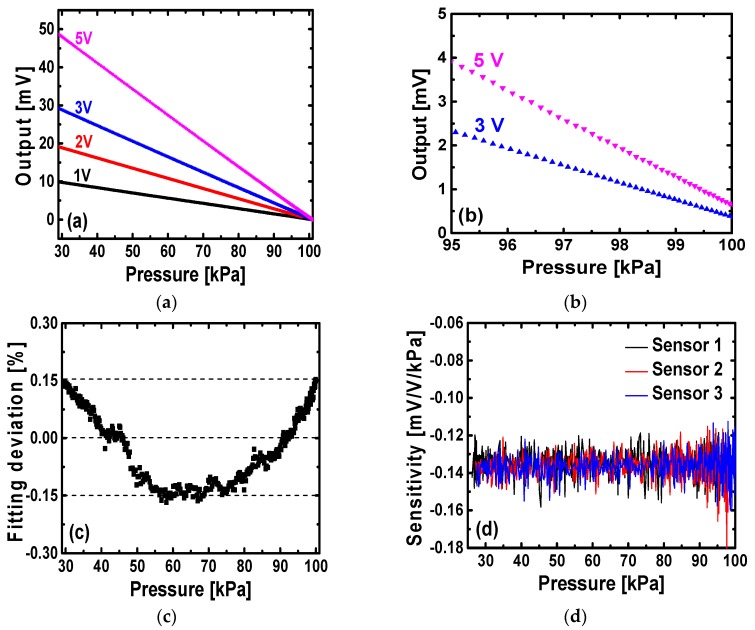
(**a**) Measured output voltages of a pressure sensing element at different input supplies; (**b**) Zoomed-in plot from 95 kPa to 100 kPa at the input voltages of 3 V and 5 V; (**c**) Linear fitting deviation of measured data at the input voltage of 1 V; (**d**) Sensitivity of three sensing elements from different locations on the wafer.

The pressure sensors are characterized in a vacuum chamber, whose pressure can be controlled by a regulating valve. A high precision Druck PACE1000 pressure indicator (General Electric, Fairfield, CT, USA) is utilized to monitor reference chamber pressures. The pressure indicator has a resolution of 6.5 Pa in the measurement range of 3.5 kPa to 130 kPa [26]. Figure 7a provides measurement results of an absolute pressure sensing element at different input voltage supplies. The sensing elements on the stretchable network have a sensitivity of 14 mV/V/bar. When pressure decreases from 100 kPa down to 30 kPa, the output voltages (in mV) increase linearly. Also, the output signals are proportional to the input voltages, as a 5 V voltage input gives five times larger output signals than does 1 V. The pressure sensor outputs illustrate a good linearity, as shown in Figure 7b, which gives zoomed-in data from 95 kPa to 100 kPa at input voltages of 3 V and 5 V. As shown, the trends between output voltages and pressures are essentially straight lines. All plotted data are raw data measured directly from multimeter outputs, without any further signal amplification, conditioning, or averaging, other than removing the output offsets. Furthermore, measured output voltage *versus* pressure at the input voltage of 1 V is fitted to a straight line with an adjusted R-square value of 0.999, and the corresponding linear fitting deviations are shown in Figure 7c. Fitting deviations are the differences between observed data and predicted values from linear regression fitting, and then are normalized by the maximum output voltage in the measured range of 30 kPa to 100 kPa. The absolute pressure sensing element has normalized fitting deviations within ±0.15%, illustrating a good linearity. In addition, the performance of three sensing elements from different locations on the wafer measured at 1 V input voltage supply are shown in Figure 7d, where sensitivity is calculated from derivative of the raw output voltage with respect to the input pressure at each individual measured point. All three sensors give a very similar pressure response and a sensitivity of ~14 mV/V/bar. Therefore, the fabricated absolute pressure sensing elements show good uniformity.

**Figure 8:**
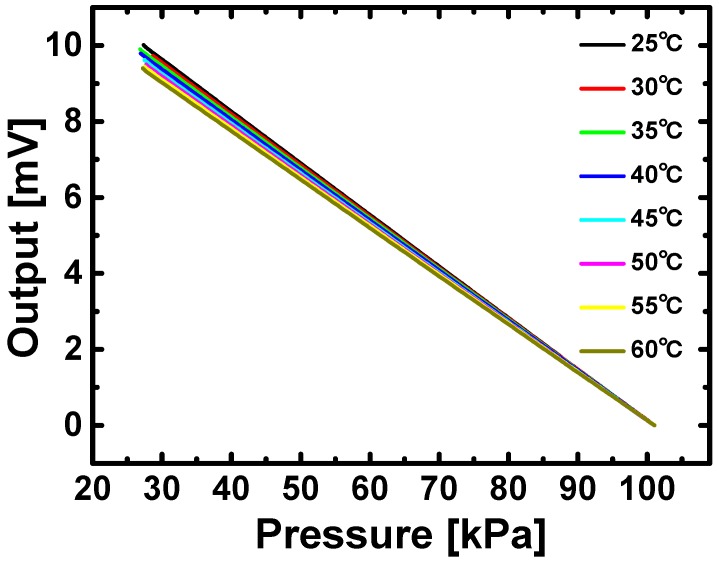
Temperature characteristics of network sensors from 25 °C to 60 °C.

Temperature characteristics are also studied. In the experiments, temperature is controlled by a model 5310 temperature controller from Arroyo Instrument (San Luis Obispo, CA, USA), which monitors platinum resistance temperature detectors and drives Peltier thermoelectric modules, simultaneously. In order to minimize measurement error, resistance temperature detectors are attached next to pressure sensors, and thermoelectric modules are placed underneath the pressure sensors. As depicted in Figure 8, the ranges of output voltages shrink linearly with the increase in temperature from 25 °C to 60 °C at the input voltage of 1 V, and the corresponding temperature coefficient of sensitivity is −0.176%/°C. This temperature coefficient can be improved on our sensing elements by further reducing the mismatch between piezoresistors and adding temperature compensation circuitry. In smart skin applications as illustrated in Figure 1, the stretchable pressure sensor network can be integrated with a temperature sensor network. Since pressure outputs can be corrected by outputs from such a reference temperature network, minuscule pressure differences can be distinguished from temperature drifts.

**Figure 9:**
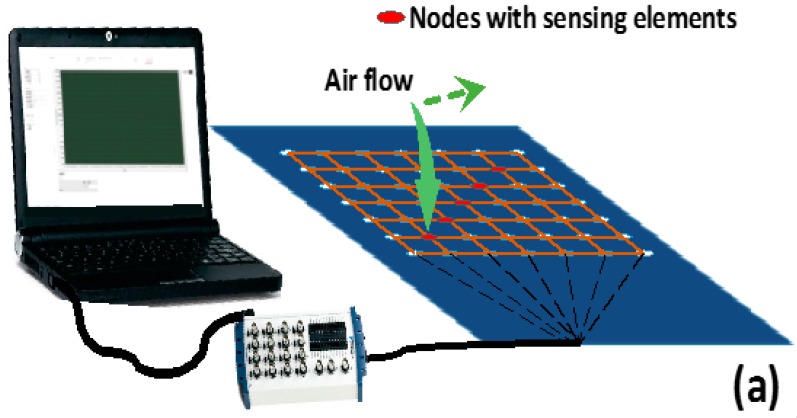

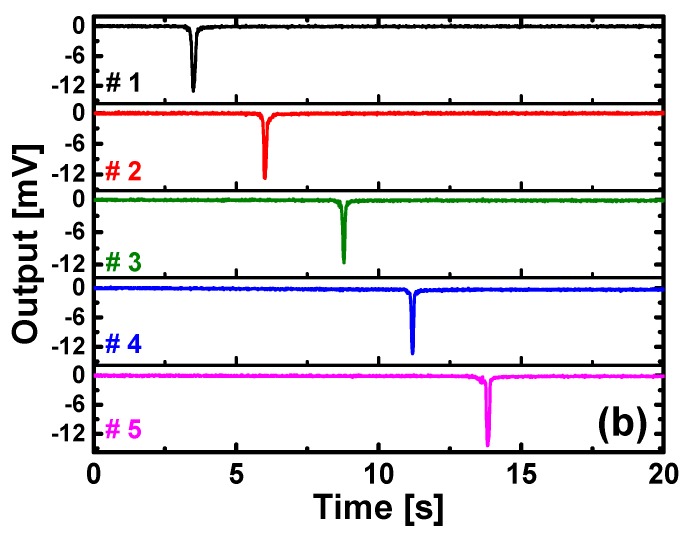
(**a**) Measurement setup of the stretchable pressure network prototype; (**b**) Network response from five nodes to a moving compressed air gun.

Figure 9a illustrates a measurement setup for the stretchable pressure network prototype. The sensor network is mounted on the surface of a flat foam board. A compressed air gun was employed in the measurement in order to apply positive pressure to the sensing nodes. As the air gun moves along the diagonal direction of the network, five sensing nodes (depicted in red) were influenced sequentially. The measured pressure data were collected by a National Instrument data acquisition board and then sent to a desktop computer for signal processing. Figure 9b depicts a network response of five nodes to the moving compressed air gun. In this measurement, the air gun, with a tiny outlet size of less than 2 mm in diameter, has a volumetric flow rate around 100 standard cubic feet per hour (SCFH). Five downward peaks represent the pressure increase on top of sensing elements caused by the compressed air gun flow in the vertical direction. Thus, the absolute pressure network is capable of detecting pressure variations at different locations. With multiple layers of interconnecting wires, more sensing nodes can be built into one network, resulting in a more accurate absolute pressure contour on smart skins. The output data can have very broad applications. For example, lift force may be directly calculated by the absolute pressure distribution data over airfoil surfaces, which holds great significance in flight monitoring and stall detection for self-awareness UAVs. Further study on data processing and algorithms for various applications is ongoing.

## 4. Conclusions

In summary, a bio-inspired stretchable absolute pressure sensor network with benefits of good performance and cost effectiveness has been investigated. This sensor network is flexible and thin, and can be attached to diverse surfaces to measure absolute pressure distribution without disturbing the airflows. Preliminary measurements have been performed on the prototype pressure network to demonstrate its excellent performance. Therefore, the stretchable absolute pressure sensor network is a promising technology toward building smart functional skins for autonomous vehicles.

## References

[1] Lanzara G., Salowitz N., Guo Z., Chang F.K. (2010). A spider-web-like highly expandable sensor network for multifunctional materials. Adv. Mater..

[2] Hammock M.L., Chortos A., Tee B.C., Tok J.B., Bao Z. (2013). 25th anniversary article: The evolution of electronic skin (e-skin): A brief history, design considerations, and recent progress. Adv. Mater..

[3] Shih W.P., Tsao L.C., Lee C.W., Cheng M.Y., Chang C., Yang Y.J., Fan K.C. (2010). Flexible temperature sensor array based on a graphite-polydimethylsiloxane composite. Sensors.

[4] Katragadda R.B., Xu Y. (2008). A novel intelligent textile technology based on silicon flexible skins. Sens. Actuators A Phys..

[5] Salowitz N., Guo Z., Li Y.H., Kim K., Lanzara G., Chang F.K. (2012). Bio-inspired stretchable network-based intelligent composites. J. Compos. Mater..

[6] Callegari S., Zagnoni M., Golfarelli A., Tartagni M., Talamelli A., Proli P., Rossetti A. (2006). Experiments on aircraft flight parameter detection by on-skin sensors. Sens. Actuators A Phys..

[7] Que R., Zhu R. (2012). Aircraft Aerodynamic Parameter Detection Using Micro Hot-Film Flow Sensor Array and BP Neural Network Identification. Sensors.

[8] Zagnoni M., Golfarelli A., Callegari S., Talamelli A., Bonora V., Sangiorgi E., Tartagni M. (2005). A non-invasive capacitive sensor strip for aerodynamic pressure measurement. Sens. Actuators A Phys..

[9] Kottapalli A.G.P., Asadnia M., Miao J.M., Barbastathis G., Triantafyllou M.S. (2012). A flexible liquid crystal polymer mems pressure sensor array for fish-like underwater sensing. Smart Mater. Struct..

[10] Mohamed A., Watkins S., Clothier R., Abdulrahim M., Massey K., Sabatini R. (2014). Fixed-wing mav attitude stability in atmospheric turbulence—Part 2: Investigating biologically-inspired sensors. Prog. Aerosp. Sci..

[11] Narducci M., Yu-Chia L., Fang W., Tsai J. (2013). CMOS MEMS capacitive absolute pressure sensor. J. Micromech. Microeng..

[12] Mohammed A.A., Moussa W.A., Lou E. (2011). High-performance piezoresistive mems strain sensor with low thermal sensitivity. Sensors.

[13] Fragiacomo G., Reck K., Lorenzen L., Thomsen E.V. (2010). Novel designs for application specific mems pressure sensors. Sensors.

[14] Lim H.C., Schulkin B., Pulickal M.J., Liu S., Petrova R., Thomas G., Wagner S., Sidhu K., Federici J.F. (2005). Flexible membrane pressure sensor. Sens. Actuators A Phys..

[15] Ahmed M., Butler D.P., Celik-Butler Z. Mems absolute pressure sensor on a flexible substrate. Proceedings of the 2012 IEEE 25th International Conference on Micro Electro Mechanical Systems (MEMS).

[16] Zhou L., Jung S., Brandon E., Jackson T.N. (2006). Flexible substrate micro-crystalline silicon and gated amorphous silicon strain sensors. Electron Devices IEEE Trans..

[17] Wang Y.-H., Lee C.-Y., Chiang C.-M. (2007). A mems-based air flow sensor with a free-standing micro-cantilever structure. Sensors.

[18] Greenwood J.C. (1988). Silicon in mechanical sensors. J. Phys. E Sci. Instrum..

[19] Eaton W.P., Smith J.H. (1997). Micromachined pressure sensors: Review and recent developments. Smart Mater. Struct..

[20] Ko W.H., Wang Q. (1999). Touch mode capacitive pressure sensors. Sens. Actuators A Phys..

[21] Barlian A.A., Park W.-T., Mallon J.R., Rastegar A.J., Pruitt B.L. (2009). Review: Semiconductor piezoresistance for microsystems. IEEE Proc..

[22] Hopcroft M.A., Nix W.D., Kenny T.W. (2010). What is the young’s modulus of silicon?. Microelectromech. Syst. J..

[23] Tian B., Zhao Y., Jiang Z., Zhang L., Liao N., Liu Y., Meng C. (2009). Fabrication and structural design of micro pressure sensors for tire pressure measurement systems (TPMS). Sensors.

[24] Kanda Y., Yasukawa A. (1997). Optimum design considerations for silicon piezoresistive pressure sensors. Sens. Actuators A Phys..

[25] Peng C.-T., Lin J.-C., Lin C.-T., Chiang K.-N. (2005). Performance and package effect of a novel piezoresistive pressure sensor fabricated by front-side etching technology. Sens. Actuators A Phys..

[26] Chiang C.-F., Graham A.B., Lee B.J., Chae H.A., Ng E.J., O’Brien G.J., Kenny T.W. Resonant pressure sensor with on-chip temperature and strain sensors for error correction. Proceedings of the 2013 IEEE 26th International Conference on Micro Electro Mechanical Systems (MEMS).

